# Genome-Wide Identification of the *Gossypium hirsutum* NHX Genes Reveals That the Endosomal-Type *GhNHX4A* Is Critical for the Salt Tolerance of Cotton

**DOI:** 10.3390/ijms21207712

**Published:** 2020-10-18

**Authors:** Wenyu Ma, Zhongying Ren, Yang Zhou, Junjie Zhao, Fei Zhang, Junping Feng, Wei Liu, Xiongfeng Ma

**Affiliations:** 1 State Key Laboratory of Cotton Biology, Institute of Cotton Research of the Chinese Academy of Agricultural Sciences, Anyang 455000, China; 82101172178@caas.cn (W.M.); renzhongying@caas.cn (Z.R.); zhaojunjie@caas.cn (J.Z.); zhangfei@caas.cn (F.Z.); 2 Hainan Key Laboratory for Biotechnology of Salt Tolerant Crops, College of Horticulture, Hainan University, Haikou 570228, China; zhouyang@hainanu.edu.cn; 3 Collaborative Innovation Center of Henan Grain Crops, Agronomy College, Henan Agricultural University, Zhengzhou 450002, China; 18838916993@163.com

**Keywords:** *Gossypium hirsutum*, salt stress, Na^+^/H^+^ antiporter, genome-wide identification, virus-induced gene silencing

## Abstract

Soil salinization, which is primarily due to excessive Na^+^ levels, is a major abiotic stress adversely affecting plant growth and development. The Na^+^/H^+^ antiporter (NHX) is a transmembrane protein mediating the transport of Na^+^ or K^+^ and H^+^ across the membrane to modulate the ionic balance of plants in response to salt stress. Research regarding NHXs has mainly focused on the vacuolar-type NHX family members. However, the biological functions of the endosomal-type NHXs remain relatively uncharacterized. In this study, 22 NHX family members were identified in *Gossypium hirsutum*. A phylogenetic analysis divided the *GhNHX* genes into two categories, with 18 and 4 in the vacuolar and endosomal groups, respectively. The chromosomal distribution of the NHX genes revealed the significant impact of genome-wide duplication during the polyploidization process on the number of *GhNHX* genes. Analyses of gene structures and conserved motifs indicated that *GhNHX* genes in the same phylogenetic cluster are conserved. Additionally, the salt-induced expression patterns confirmed that the expression levels of most of the *GhNHX* genes are affected by salinity. Specifically, in the endosomal group, *GhNHX4A* expression was substantially up-regulated by salt stress. A yeast functional complementation test proved that *GhNHX4A* can partially restore the salt tolerance of the salt-sensitive yeast mutant AXT3. Silencing *GhNHX4A* expression decreased the resistance of cotton to salt stress because of an increase in the accumulation of Na^+^ in stems and a decrease in the accumulation of K^+^ in roots. The results of this study may provide the basis for an in-depth characterization of the regulatory functions of NHX genes related to cotton salt tolerance, especially the endosomal-type *GhNHX4A*. Furthermore, the presented data may be useful for selecting appropriate candidate genes for the breeding of new salt-tolerant cotton varieties.

## 1. Introduction

Plant growth is influenced by a variety of biotic factors (e.g., pathogens and insect pests) and abiotic factors (e.g., salt, drought, cold, and osmotic stresses) [1,2]. Salt stress, which is one of the most serious abiotic stresses, can significantly limit crop production [3,4]. Increases in the soil Na^+^ concentration inhibit crop growth and development and can even lead to plant death [5]. Soil salinization, which is a global environmental problem caused by human activities, severely restricts agricultural productivity [6]. The soil salinization of almost 20% of the irrigated agricultural land is nearing harmful levels, and this figure is increasing on a yearly basis [7]. Therefore, there is an urgent need for research regarding crop salt tolerance, especially pioneer crops, so that new varieties adapted to saline environments can be developed and cultivated.

The response of plants to salt can be divided into three ways: refusing to absorb salt, reducing the salt concentration in the cell, and actively accumulating some small molecular organic compounds and protein protective agents [8,9,10,11,12,13]. More precisely, salinity tolerance mainly involves an adaptation to osmotic stress, Na^+^ exclusion from the cytoplasm, tolerance to Na^+^ and Cl^−^ accumulation, and compartmentalization, all of which occur in a coordinated manner [6]. The compartmentalization of Na^+^ into appropriate cell compartments driven by the Na^+^/H^+^ antiporter (NHX) is an effective mechanism for plant salt tolerance [14]. Studies have shown that under salt stress, plants use the NHXs to partition Na^+^ into vacuoles, which can prompt cells to absorb water from the external stress environment to maintain osmotic balance, reduce the toxic effects of salt ions on organelles in the cytoplasm, and adjust the pH and Na^+^ concentration in the cytoplasm [15,16]. Therefore, NHX is especially important for salt tolerance.

The NHX is a type of transmembrane antiporter, belonging to the cation/proton antiporter-1 gene family. Plant NHXs can be divided into two classes based on subcellular localization [17,18,19]. In *Arabidopsis thaliana*, *AtNHX1–4*, which belong to the first NHX subfamily (Class 1), are localized in the vacuolar membrane [20]. Research on the salt and drought resistance mechanism of plants shows that they provide energy through the transmembrane proton gradient formed by H^+^-ATPase or H^+^-PPase at the vacuolar membrane, and mediate Na^+^ regionalization to minimize the cellular damage due to excessive amounts of salt ions and stabilize the osmotic pressure of cells so that plants can adapt to the salty and drought environment [21,22,23,24]. Previous studies proved that the NHX proteins of the Class 1 subfamily can be significantly induced by salt stress and can increase the tolerance of yeast and plants to Na^+^ [25,26,27]. Additionally, studies have shown that after *AtNHX1* is overexpressed in *Arabidopsis*, transgenic plants can grow and develop normally under 200 mmol/L NaCl treatment [16]. Similarly, overexpressed *AtNHX1* gene in *Actinidia deliciosa*, the transgenic plants can resist 200 mmol/L NaCl stress. Compared with wild-type plants, the transgenic lines have increased NHX protein activity, enhanced osmotic regulation and antioxidant capacity, accumulated more Na^+^, and significantly improved yield [28]. The overexpression of NHX genes can also up-regulate the expression of other stress resistance-related genes and contribute to responses to salt stress [29]. The members of the second NHX subfamily (Class 2), *AtNHX5–6*, are primarily localized in the endosomal compartments as associated with Golgi bodies and prevacuolar compartments, where they mainly regulate K^+^ and pH homeostasis under saline conditions [30]. Both *AtNHX5* and *AtNHX6* can enhance the salt and K^+^ resistance and stabilize the pH of the endosomal compartments of the AXT3 yeast mutant, which is extremely sensitive to high potassium and high salt [31]. The heterologous expression of *AtNHX5* in monocotyledonous plants, such as *Oryza sativa*, induces the accumulation of Na^+^ and K^+^ following a high-salt treatment, with transgenic plants exhibiting increased salt tolerance [32]. The localization of LeNHX2 is in the vesicles around the vacuole and the nucleus, similar to that of AtNHX5 and AtNHX6, which are located in the endosomal compartments [33]. Researchers observed that *LeNHX2*-overexpressing transgenic *A. thaliana* plants cannot grow at low K^+^ concentrations. Additionally, under salt stress conditions, their K^+^ content is significantly higher than that of wild-type plants. Accordingly, *LeNHX2* appears to mainly mediate K^+^/H^+^ transport and improve salt tolerance by maintaining cellular K^+^ levels [33]. In addition to responding to salt stress, Class 2 NHX members are also important regulators of growth and development [30].

As an important source of natural fiber, plant proteins, and edible oil, cotton is an economically valuable crop [34]. The upland cotton (*Gossypium hirsutum*), a tetraploid cotton, is the most commonly cultivated cotton species worldwide, which is derived from a polyploidization event that united A-genome diploids resembling *Gossypium arboreum* and D-genome diploids resembling *Gossypium raimondii*, approximately 1–2 million years ago (MYA) [35]. Although cotton is considered tolerant to drought conditions and moderately tolerant to high salinity, its sensitivity to these abiotic stresses varies greatly among varieties and genotypes [36,37]. Thus, salt stress can still significantly affect cotton yield and fiber quality. It has been reported that the expression of some cotton NHXs showed an upward trend in cotton under salt stress, and the expression patterns of some genes were different under low- and high-salt conditions [38]. The expression of *GhNHX1* in yeast with a mutated NHX results in functional complementation [39]. The same study proved that transgenic tobacco plants overexpressing *GhNHX1* are more salt-tolerant than wild-type plants [39]. Furthermore, transgenic cotton plants expressing *AtNHX1* reportedly produce larger biomass and more fibers when treated with 200 mM NaCl in a greenhouse compared with control plants, implying that *AtNHX1* can increase cotton salt stress tolerance [40]. Researchers found that NHX members in cotton could be involved in various developmental processes and stress responses by maintaining the turgor pressure, pH, and ionic homeostasis [41]. However, the functions of cotton endosomal-type NHX members related to Na^+^/H^+^ exchange and localization in plant cells remain unclear.

In this study, we applied bioinformatics tools to identify NHX gene family members in *G. hirsutum*. Quantitative real-time PCR (qPCR) was used to analyze the *GhNHX* expression patterns, after which the key gene related to salt tolerance among the endosomal group members was identified via yeast functional complementation and virus-induced gene silencing (VIGS). The resulting data may be relevant to future investigations regarding the utility of NHX genes for developing high-quality cotton varieties resistant to salinity and other abiotic stresses.

## 2. Results

### 2.1. Genome-Wide Identification and Phylogenetic Relationships of NHX Genes

In this study, query sequences were derived from the protein sequences encoded by *A. thaliana*, *Populus trichocarpa*, *O. sativa*, and *Zea mays* NHX genes. The BlastP and tBlastN algorithms were used to screen *G. hirsutum*, *G. raimondii*, and *G. arboreum* genome databases to obtain candidate sequences. After searching, validating, and removing redundant sequences, all candidate genes were analyzed in the Pfam database to verify the presence of NHX conserved domains (PF00999). A total of 44 members of the NHX gene family were identified in the cotton genomes (22 in *G. hirsutum* and 11 each in *G. raimondii* and *G. arboreum*). The A and D subgenomes of the tetraploid upland cotton each contained 11 genes. The 11 genes in the A subgenome were named first according to their positions (*GhNHX1A–11A*), after which the 11 genes in the D subgenome were named according to their homology to the genes in the A subgenome (*GhNHX1D–11D*) (Tables S1 and S2). Then, the NHX genes in *G. raimondii* and *G. arboreum* were named based on their homology to the *G. hirsutum* NHX genes, respectively (Tables S3 and S4).

**Figure 1:**
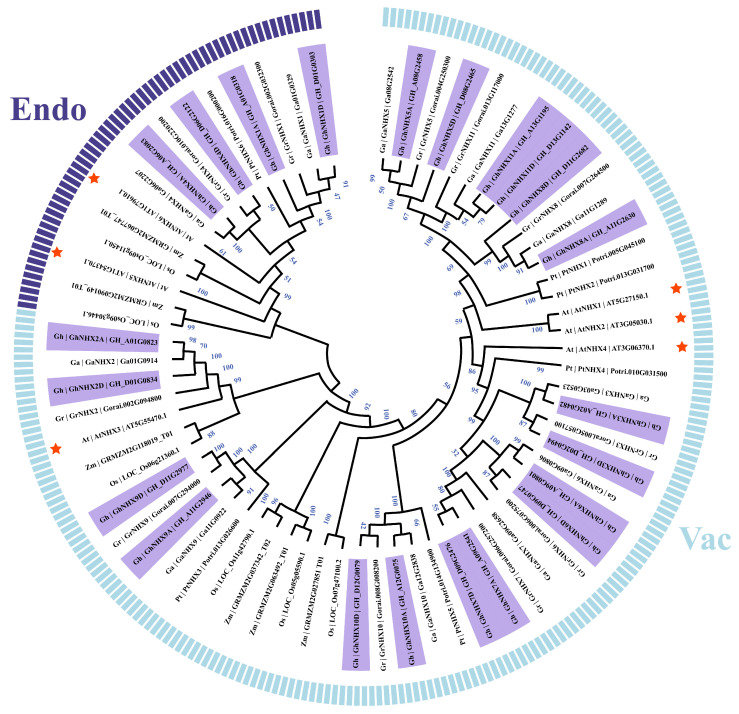
Phylogenetic analysis of the Na^+^/H^+^ antiporters (NHXs). The phylogenetic tree was generated with the neighbor-joining method of MEGA X (1000 bootstrap replicates) using 22 NHXs from *G. hirsutum* (Gh) (Table S2) and 49 NHXs from *G. arboreum* (Ga), *G. raimondii* (Gr), *A. thaliana* (At), *P. trichocarpa* (Pt), *O. sativa* (Os), and *Z. mays* (Zm) (Tables S4 and S5). The NHXs from *G. hirsutum* are presented by purple backgrounds, and the NHXs from *A. thaliana* are indicated by red stars. All NHXs are classified into one of two classes (Class 1: vacuolar-type, Class 2: endosomal-type), which are differentiated by different color arcs. Numbers on the branches are bootstrap values.

To evaluate the phylogenetic relationships of NHX genes, a phylogenetic tree was constructed based on the *G. hirsutum, G. raimondii*, *G. arboreum*, *A. thaliana, P. trichocarpa*, *O. sativa*, and *Z. mays* NHX protein sequences (Figure 1). The phylogenetic analysis combined with the results of earlier research divided the NHX proteins into the following two families: Class 1 and Class 2, which consisted of the vacuolar-type and endosomal-type NHXs, respectively. Class 1 had more members than Class 2. Additionally, Classes 1 and 2 included 18 and 4 upland cotton members, respectively.

**Figure 2:**
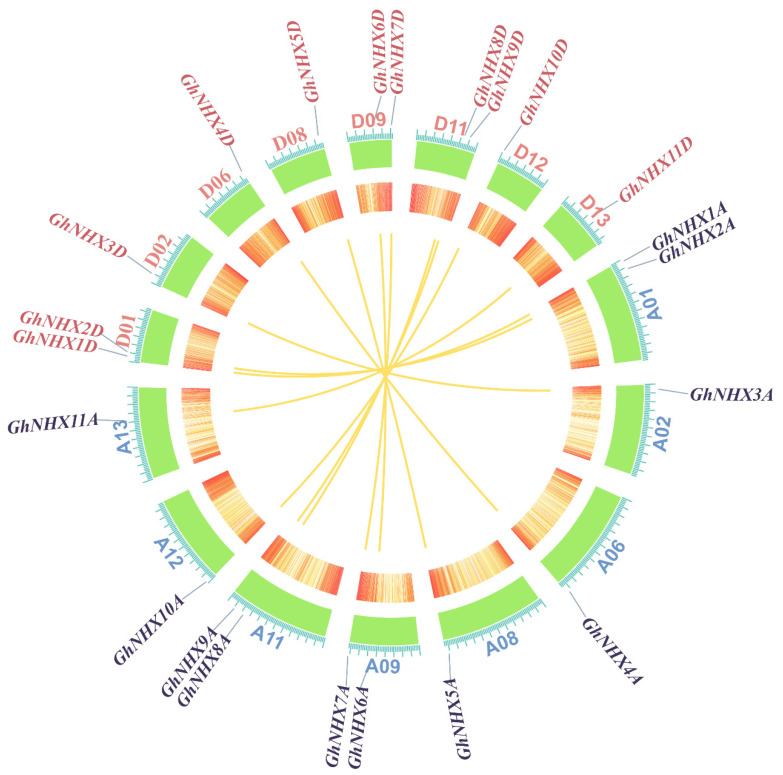
Chromosomal distribution and homologous relationships of the *GhNHX* genes. *G. hirsutum* chromosomes are presented in a circular form. The A and D subgenomes of *G. hirsutum* were labeled by blue and red fonts, respectively. Approximate positions of *GhNHX* genes are indicated with short gray lines on the circle. Homologous *GhNHX* genes between the A and D subgenomes are connected by yellow curves. The red and yellow circles in the middle represent the gene density on each chromosome, where red indicates high density and yellow indicates low density.

We identified 11 pairs of paralogous genes in the upland cotton NHX family based on the phylogenetic analysis (Figure 1). An examination of chromosomal distribution revealed that the 22 NHX genes in upland cotton were located on 16 of 26 chromosomes. Specifically, chromosomes A01, A09, A11, D01, D09, and D11 contained two members each, whereas chromosomes A02, A06, A08, A12, A13, D02, D06, D08, D12, and D13 included one member each. There was a one-to-one relationship among the homologous genes of the A and D subgenomes of upland cotton (Figure 2).

### 2.2. Gene Structures and Conserved Motifs of NHXs in G. hirsutum

**Figure 3:**
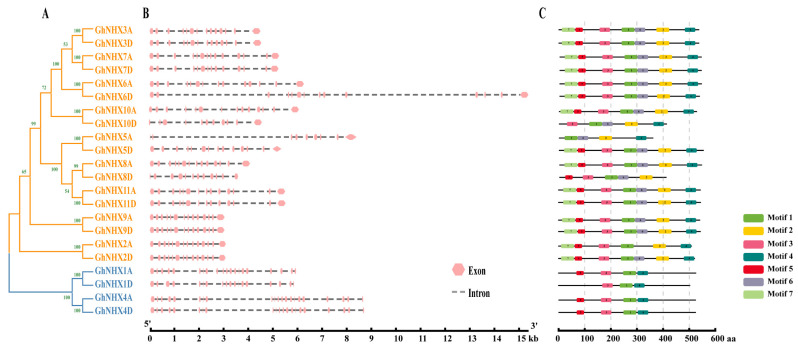
Phylogenetic relationships, gene structures, and motif architectures of *GhNHX* genes. (**A**) Phylogenetic relationships among *GhNHX* genes. The phylogenetic tree was constructed with the neighbor-joining method of MEGA X (1000 bootstrap replicates). All NHXs are classified into two classes, which are differentiated by color. The vacuolar-type members are indicated by orange branches, and the endosomal-type members are indicated by blue branches. Numbers on the branches are bootstrap values. (**B**) Exon-intron structures of *GhNHX* genes. Gene structure maps were drawn with the Gene Structure Display Server. The exons and introns are indicated by pink boxes and gray lines, respectively. (**C**) Motif compositions in proteins encoded by the *GhNHX* genes. Conserved motifs were predicted with the MEME software (http://meme-suite.org/). Motif lengths are presented proportionally.

To clarify the evolutionary relationships among the *GhNHX* gene family members, we constructed a separate phylogenetic tree (Figure 3A) and performed a comparative structural analysis (Figure 3B). We detected substantial differences in the *GhNHX* gene lengths. These genes were divided into two categories based on the number of exons. The members of Classes 1 and 2 generally consisted of 14 and 21 exons, respectively, indicative of the relatively highly conserved exon-intron structures within the same class.

We also explored the upland cotton NHX protein sequence features with the MEME program, which predicted the presence of seven motifs in the NHX proteins (Figure 3C). The motif distribution was similar among the members of the same subgroup. Most proteins had motifs 1 and 4, suggesting these motifs have crucial roles. Motifs 2 and 6 were exclusive to the vacuolar-type cluster. The diversity in the motifs among the NHX subclasses implied that the functions of these proteins may have changed during evolution. 

### 2.3. Expression Patterns of GhNHX Genes under a Salt Treament

**Figure 4:**
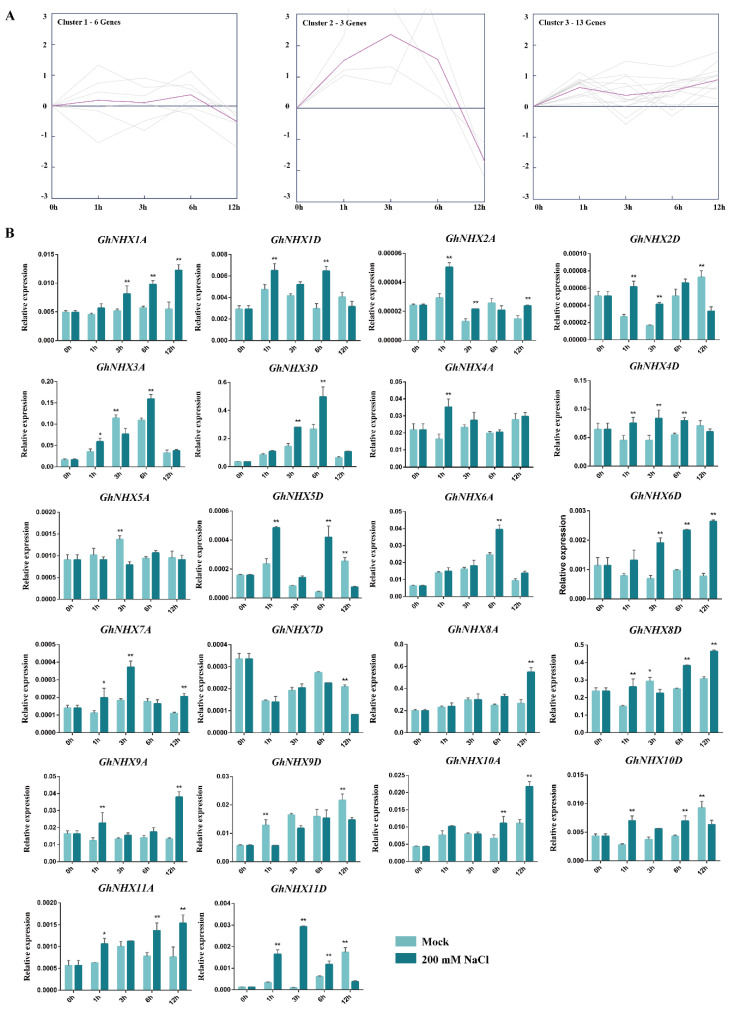
Expression patterns of *GhNHX* genes in leaves after the 200 mM NaCl treatment. (**A**) Cluster analysis of expression profiles of *GhNHX* genes under the NaCl treatment. The cluster analysis was developed using the K-means method on the expression profiles of *G. hirsutum* NHX genes under the NaCl treatment. The expression pattern of each gene is indicated by a gray line, and the representative expression pattern of each cluster is represented by a purple line. The x-axis presents the examined time-points after the salt treatment, whereas the y-axis presents the scale of the relative expression levels. (**B**) Expression profiles of *GhNHX* genes under the NaCl treatment. The x-axis presents the examined time-points after the salt treatment, whereas the y-axis presents the relative expression levels. Error bars indicate the standard deviation of three biological replicates (* *p* < 0.05, ** *p* < 0.01; *t*-test).

Previous studies confirmed that NHXs could be induced by salt treatments and have key functions related to the salt tolerance of various plant species. Thus, expression patterns of *GhNHX* genes under the 200 mM NaCl treatment were analyzed (Figure 4). As shown in Figure 4A, there were essentially three types of expression profiles based on the cluster analysis. Six genes including *GhNHX1D, GhNHX4D, GhNHX5A, GhNHX7D, GhNHX9D,* and *GhNHX10D* fell into the Cluster 1, only three genes including *GhNHX2D*, *GhNHX5D,* and *GhNHX11D* in the Cluster 2, while 13 genes including *GhNHX1A, GhNHX2A, GhNHX3A, GhNHX3D, GhNHX4A, GhNHX6A, GhNHX6D, GhNHX7A, GhNHX8A, GhNHX8D, GhNHX9A, GhNHX10A*, and *GhNHX11A* in the Cluster 3. We further analyzed the salt stress expression pattern of each *GhNHX* (Figure 4B). In the Cluster 1 genes, *GhNHX1D* and *GhNHX10D* were significantly up-regulated under salt stress at 1 and 6 h, and the up-regulated expression of *GhNHX4D* occurred at 1, 3, and 6 h after the salt treatment, while *GhNHX1D* and *GhNHX4D* were slightly down-regulated at 12 h and *GhNHX10D* was significantly down-regulated at 12 h. In addition, the expression of *GhNHX5A*, *GhNHX7D,* and *GhNHX9D* were usually down-regulated or not significantly affected by salt treatment. In the Cluster 2 genes, *GhNHX2D*, *GhNHX5D,* and *GhNHX11D* occurred rapidly after the salt treatment, while they all were significantly down-regulated until 12 h. In the Cluster 3 genes, the expression levels were induced by salt treatment at most time points, while just *GhNHX3A* and *GhNHX8D* were significantly down-regulated at 3 h. Our results reflect the salt stress response of cotton NHXs.

### 2.4. Functional Complementation of GhNHX4A in Yeast Mutants

**Figure 5:**
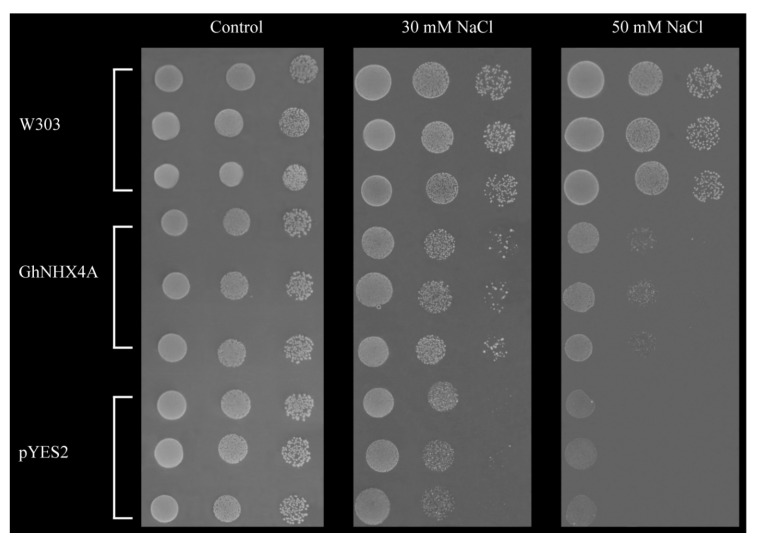
Functional complementation of *GhNHX4A* in yeast mutants. W303: Wild-type strain W303; GhNHX4A: Mutant strain AXT3 transformed with pYES2-*GhNHX4A*; pYES2: Mutant strain AXT3 transformed with empty pYES2.

According to the results of gene expression analysis, among the four endosomal-type NHX genes, *GhNHX1A* and *GhNHX4A*, which were considerably up-regulated by salt stress and had a relatively high abundance expression level, were selected to conduct functional complementary experiments in yeast mutants. Yeast mutant strain AXT3 separately transformed with pYES2-*GhNHX1A*, pYES2-*GhNHX4A* and empty pYES2, and wild-type yeast strain W303 (control) were treated with two NaCl concentrations (Figure 5 and Figure S1). Because it lacks the main endogenous Na^+^ transporter, the growth of yeast strain AXT3 transformed with pYES2 was inhibited by 30 mM NaCl. However, yeast strain AXT3 transformed with *GhNHX1A* and yeast strain AXT3 transformed with *GhNHX4A* were more tolerant to salt stress, likely because of a partially recovered Na^+^ transport function, and was still able to grow on medium containing 50 mM NaCl. Moreover, yeast strain AXT3 transformed with *GhNHX4A* was more tolerant to salt stress than with *GhNHX1A*. Therefore, *GhNHX4A* was selected for further analyses.

### 2.5. Subcellular Localization of GhNHX4A

**Figure 6:**
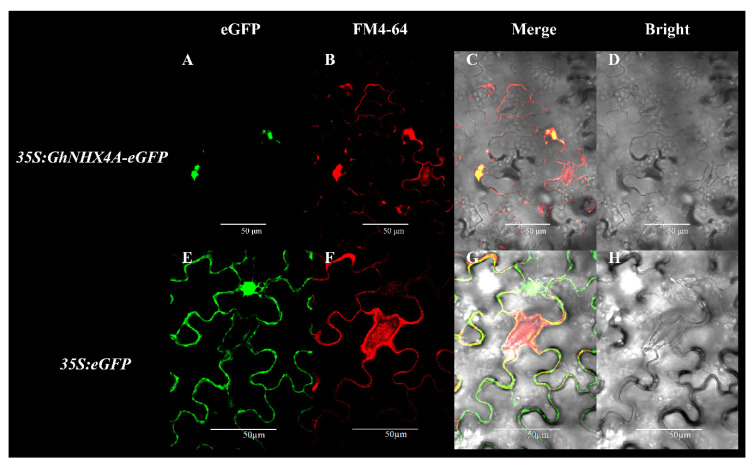
Subcellular localization of GhNHX4A in tobacco leaves. (**A**–**D**) *35S:GhNHX4A-eGFP*, eGFP-tagged GhNHX4A; (**E**–**H**) *35S:eGFP*, control; (**A**,**E**) Green fluorescence image; (**B**,**F**) Red fluorescence stained by FM4-64; (**C**,**G**) Red fluorescence, green fluorescence and bright field images are merged; (**D**,**H**) Bright field image.

Protein functions are closely related to their localization. The evolutionary analysis indicated that GhNHX4A is likely localized to the endosomal compartment (Figure 1). Therefore, we constructed the *35S:GhNHX4A-eGFP* expression vector, which was subsequently transiently expressed in tobacco leaves, with *35S:eGFP* used as the control. When expressing eGFP alone, the green fluorescence was observed throughout the plasma membrane, cytoplasm, and nucleus with no specificity (Figure 6E). While expressing GhNHX4A-eGFP, the green fluorescence appeared in the vesicle-like structures around the plasma membrane, vacuolar membrane, and nucleus, but it was not certain whether it was located in the endosomal compartment (Figure 6A). Therefore, we continue to use a maker dye FM4-64. Studies have shown that colocalization of proteins with internalized FM4-64 may be used to judge whether a particular compartment labeled by a specific protein is a putative endosomal compartment in plant cells [42]. After staining with FM4-64, the green fluorescence of eGFP, except for the nucleus and other structures, is superimposed with red fluorescence to form yellow fluorescence (Figure 6G). The vesicle-like green fluorescence of GhNHX4A-eGFP overlaps with the red fluorescence to show yellow fluorescence (Figure 6C), indicating that GhNHX4A is located in the endosomal compartment.

### 2.6. Virus-Induced Gene Silencing of GhNHX4A in G. hirsutum

**Figure 7:**
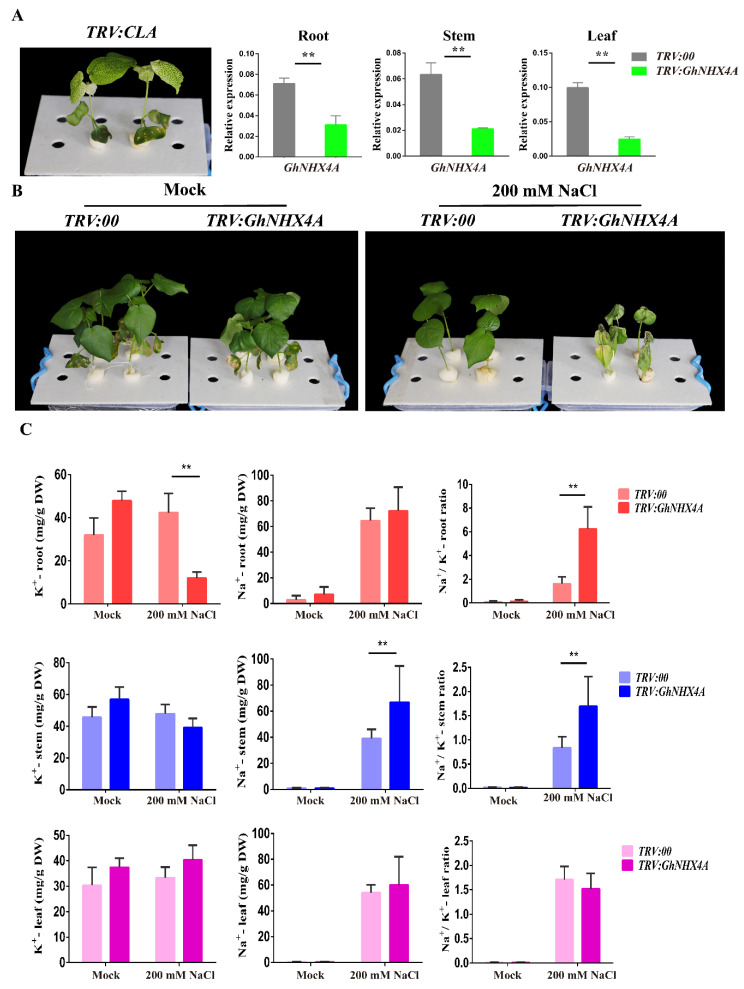
Functional analysis of *GhNHX4A* in cotton response to salt stress. (**A**) Gene silencing efficiency of *TRV:00* and *TRV:GhNHX4A*. *TRV:CLA* was used as the positive control, and *GhHIS3* served as the internal reference gene. Error bars indicate the standard deviation of at least three biological replicates (** *p* < 0.01; *t*-test). (**B**) Representative images of *TRV:00* and *TRV:GhNHX4A* plants treated with water (mock) or 200 mM NaCl. (**C**) Na^+^ and K^+^ contents and Na^+^/K^+^ ratios of the *TRV:00* and *TRV:GhNHX4A* plants treated with water (mock) or NaCl. Red, blue, and purple refer to the roots, stems, and leaves, respectively. Error bars indicate the standard deviation of at least three biological replicates (** *p* < 0.01; *t*-test).

To precisely characterize the *GhNHX4A* functions related to cotton salt tolerance, we conducted a VIGS experiment. An *Agrobacterium tumefaciens*-mediated transformation method was used to insert *TRV:00* (empty VIGS vector) and *TRV:GhNHX4A* into the cotyledons of 10-day-old upland cotton (TM-1) seedlings. After 12 days (i.e., when the leaves of the positive control plants appear albino phenotype), roots, stems, and leaves were cut from cotton plants. Total RNA was extracted and then reverse-transcribed to synthesize cDNA. Finally, the silencing of *GhNHX4A* expression was confirmed by qPCR, and the level of *GhNHX4A* expression in roots, stems, and leaves of the *TRV:GhNHX4A* plants remarkably decreased compared with the *TRV:00* plants (Figure 7A). To study the silencing specificity, the expression of *GhNHX4D*, as the homologous gene of *GhNHX4A*, was also analyzed by qPCR, and no significant difference was observed between the *TRV:00* and *TRV:GhNHX4A* plants (Figure S2). The results revealed strong and specific silencing of *GhNHX4A* in the *GhNHX4A*-silenced plants.

The *TRV:00* and *TRV:GhNHX4A* plants were simultaneously treated with 200 mM NaCl. Phenotypic changes were observed at 10 days after the treatment (Figure 7B). Compared with the *TRV:00* plants, the *TRV:GhNHX4A* plants were more sensitive to salt stress, implying this gene contributes to the salt tolerance of cotton.

In the current study, the Na^+^ and K^+^ contents in the *TRV:00* and *TRV:GhNHX4A* plants were determined by atomic absorption spectroscopy (Figure 7C). There were no significant differences in the K^+^ and Na^+^ contents or the Na^+^/K^+^ ratio of the roots, stems, and leaves between the *TRV:00* and *TRV:GhNHX4A* plants in the mock group. Additionally, no significant differences were also found in the K^+^ and Na^+^ contents or the Na^+^/K^+^ ratio between the *TRV:00* and *TRV:GhNHX4A* leaves under the 200 mM NaCl treatment. However, in contrast, the Na^+^ content was significantly higher in the *TRV:GhNHX4A* stems than in the *TRV:00* stems, whereas the K^+^ content was significantly lower in the *TRV:GhNHX4A* roots than in the *TRV:00* roots under the 200 mM NaCl treatment. Moreover, following the 200 mM NaCl treatment, the Na^+^/K^+^ ratio was significantly higher in the *TRV:GhNHX4A* roots and stems than in the corresponding *TRV:00* tissues. Obviously, *GhNHX4A* in the cotton stems is mainly involved in the accumulation of Na^+^, whereas in the roots, it controls the cellular accumulation of K^+^. Accordingly, *GhNHX4A* helps mediate the Na^+^ and K^+^ exchange in cotton, but its function may vary among tissues.

## 3. Discussion

### 3.1. Cotton NHX Gene Family

Salt stress leads to an ionic imbalance in plant cells, and the resulting toxic cellular ion concentrations lead to ion stress [6]. Therefore, ion homeostasis has important regulatory effects on plant growth and development, with the associated cation/proton antiporters crucial for stabilizing ion levels in plants [14,19,43]. The plant NHX genes belong to a large family of monovalent cation/proton antiporter-1 (CPA1) [17]. All plant NHXs can be divided into two subclasses, with Class 1 NHXs localized to the vacuolar membrane and Class 2 NHXs localized to the endosomal compartment [20].

In this study, 22 *G. hirsutum* NHX genes were identified, which is more than the number of NHX genes in other species [20,44,45,46]. Thus, the upland cotton NHX gene family expanded substantially during evolution. A phylogenetic analysis indicated that upland cotton NHXs can be divided into two subcategories, consistent with the results of earlier research [20]. Moreover, our phylogenetic tree revealed that the NHX genes of upland cotton and dicotyledons (*A. thaliana* and *P. trichocarpa*) and monocotyledon (*O. sativa* and *Z. mays*) are clustered in the two branches, suggesting these two subfamilies existed before the formation of monocotyledons and dicotyledons.

Gene duplication is a common phenomenon in plants [47,48,49]. Specific molecular mechanisms regulate different gene duplication events, including tandem duplications and recombination or DNA replications and whole-genome duplication [50,51]. The evolution of duplicate genes and the subsequent differentiation provided the original genetic resources for adaptive evolution, while also contributing to the development of new genes [50,51]. An analysis of the cotton genome revealed obvious NHX gene duplications. Specifically, 11 NHX family genes were identified in the A genome of *G. arboreum* as well as in the D genome of *G. raimondii*. Moreover, the 11 members of the upland cotton A subgenome correspond to the 11 members of the D subgenome. Therefore, the upland cotton NHX genes may have resulted primarily from genome-wide duplication during the polyploidization event, which could increase the functional diversity of the NHXs.

Both *GhNHX1A/1D* and *GhNHX4A/4D* are structurally significantly different from the other NHX family members, which is consistent with the results of the phylogenetic analysis, confirming that the *GhNHX* family members form two subgroups. The vacuolar-type members of different species have between 13 and 14 exons, whereas the endosomal-type NHX genes have 18–22 exons [44,46,52,53,54]. The results of the current study are consistent with these published findings, implying that the important functions of this family were conserved during evolution. Notably, *GhNHX5A* and *GhNHX5D* are homologs in different subgenomes of *G. hirsutum*. Interestingly, *GhNHX5D* has a classical gene structure, whereas *GhNHX5A* has fewer exons. These observations are indicative of the independent evolutionary events that affected some cotton NHX genes.

### 3.2. Expression Profiles of GhNHX Genes under the Salt Treatment

There has been considerable interest in the roles of NHX genes among researchers investigating plant salt tolerance, with previous studies indicating that plant NHXs can respond to various environmental stresses and hormone treatments [55,56]. In an earlier study, the Km values (for Na^+^) of the sunflower NHXs on the root vacuolar membrane microcapsules were 64 and 8 mM for plants treated with 75 and 150 mM NaCl, respectively, implying that salt stress can induce the post-transcriptional modification of NHX and enhance sunflower tolerance to high salinity [57]. In the salt-tolerant cotton strain ZM3, an exposure to saline conditions reportedly leads to *GhNHX1* expression levels that are 3 and 7 times higher than that of the salt-sensitive strains ZMS17 and ZMS12, respectively. Moreover, salt stress and ABA stress can substantially up-regulate *GhNHX1* expression in cotton seeds [39]. The results of the current study indicate that the expression of almost all upland cotton NHX genes is induced by salinity stress, but to varying degrees. This suggests that although all *GhNHX* genes are involved in upland cotton salt stress responses, different stress levels may differentially affect the expression of these genes. This transcriptional diversity may be determined by the respective genetic structures as well as physical and chemical properties.

In poplar seedlings under salt stress conditions, the expression levels of two *PtNHX* genes are significantly up-regulated at 12 h after treatments [46]. In the current study, the expression levels of several Cluster 3 genes, such as *GhNHX1A*, increased as the duration of the salt treatment increased, with peak levels at 12 h. A 200 mM NaCl treatment was observed to significantly increase the root transcription levels of all *PeNHX* genes at 6 h [58], which is also the time-point when the expression of Cluster 1 genes (e.g., *GhNHX3D*) peaked in our study. The expression of the Cluster 3 genes, such as *GhNHX4A*, changed quickly in response to the salt treatment. Among *A. thaliana* genes, *AtNHX5* is the most homologous to *GhNHX4A*, and its expression is induced by NaCl, but not by sorbitol or exogenous ABA, reflecting the specificity of its response to ion stress [20]. In another study, the *BrNHX7* expression level was not significantly influenced by salinity and remained low following a salt treatment [59]. Similarly, we observed that the expression of some genes, such as *GhNHX7D*, was unaffected in the early stage of salt stress, suggesting these genes may not contribute substantially to the physiological responses associated with salt tolerance.

### 3.3. GhNHX4A-Mediated Salt Tolerance

Cotton is a salt-tolerant crop, but long-term exposure to high salt concentrations can significantly affect cotton growth as well as fiber yield and quality [60,61]. Because the mechanism underlying Na^+^ detoxification in yeast is similar to that in plants, yeast cells may be applicable for functional complementation experiments designed to clarify the roles of plant NHXs in salt stress responses [62]. In this study, *GhNHX4A* was transformed into the salt-sensitive yeast mutant strain AXT3 to assess the recovery of salt resistance. We observed that the salt tolerance of the yeast mutant cells transformed into with *GhNHX4A* was restored. Specifically, cells expressing *GhNHX4A* grew well in medium supplemented with 50 mM NaCl, in contrast to the significantly inhibited growth of the AXT3 cells lacking the transgene. Similar to *GhNHX4A*, *TaNHX2*, which is located in the endosomal compartment, is responsive to salt stress and can complement the salt sensitivity of yeast mutants [26].

We examined the subcellular localization of GhNHX4A in tobacco leaves transformed with an *A. tumefaciens*-mediated method. In tobacco leaf epidermal cells, GhNHX4A was detected in the endosomal compartments of cells, which was consistent with the results of the phylogenetic analysis as well as the findings of earlier studies involving the mapping of homologous genes in *A. thaliana* and tomato [30,63,64].

The absence of endosomal-type NHXs makes seed germination and seedling growth extremely sensitive to salt stress [30]. In our VIGS experiment, the silencing of *GhNHX4A* resulted in decreased salt tolerance. The ion content of cells is closely related to the salt tolerance of plants, and increases in ion concentrations to toxic levels will decrease the salt tolerance of plants [6,65]. Thus, in this study, we measured the K^+^ and Na^+^ contents in the roots, stems, and leaves of the *GhNHX4A*-silenced plants to further elucidate the salt tolerance regulated by *GhNHX4A*. The result suggests that the *TRV:GhNHX4A* plants accumulate Na^+^ in the stem, but not K^+^ in the roots, in response to salt stress. The overexpression of *AtNHX1* in *A. thaliana* increases the resistance of the transgenic plants to Na^+^ and induces the absorption of K^+^ [16,66,67]. Similarly, *AtNHX1*-overexpressing wheat plants treated with 100–150 mM NaCl have lower Na^+^ concentrations and higher K^+^ concentrations than wild-type lines [68]. *HvNHX1* was isolated from barley based on the sequence of *AtNHX1* and *OsNHX1*, which can transport Na^+^ and K^+^, and improve the salt tolerance of plants [69]. These studies provide evidence that NHX proteins can localize Na^+^ or K^+^ in cells to minimize ion toxicity.

Research on endosomal-type NHXs proved that the overexpression of *AtNHX5* in *A. thaliana* can enhance seed germination and the salt tolerance of seedlings. Specifically, the dry and fresh weights as well as the Na^+^ and K^+^ contents in shoots are reportedly higher for *AtNHX5*-overexpressing plants than for wild-type plants under saline conditions [70]. Additionally, *AtNHX5* can not only increase the accumulation of Na^+^ in transgenic *Torenia fournieri* leaves, but it can also significantly weaken the adverse effects of salt treatment on the K^+^ content in leaves. Accordingly, *AtNHX5* is useful for improving the salt tolerance of *Torenia* species because of its effects on the enrichment of Na^+^ and K^+^ via the antiporter [71]. *PeNHX6* encodes Na^+^ and K^+^ transporters, whereas the transporter encoded by *AtNHX6* mainly mediates K^+^/H^+^ transport, and only weakly controls Na^+^/H^+^ transport [31,58]. Therefore, the ion transport functions of NHX6 vary among species [31]. These results indicate that endosomal-type NHXs, like vacuolar-type NHXs, help regulate Na^+^ and K^+^ homeostasis, and the mechanism underlying their ion transport may be similar. We speculate that *GhNHX4A* can partition some of the Na^+^ ions in the endosomal compartment in cotton stems, thereby limiting the inhibitory effects of salt stress on cotton growth. In the roots, K^+^/H^+^ transport is induced, and salt tolerance is improved by maintaining the cellular abundance of K^+^. In a previous study, the silencing of *LeNHX2* in tomato inhibited K^+^ accumulation and weakened the salt tolerance of plants [33], which is consistent with the results of our study. Accordingly, *GhNHX4A* has important functions related to plant resistance to salt stress.

## 4. Materials and Methods 

### 4.1. Identification of NHX Genes in Gossypium Species

To identify the NHX genes in *G. hirsutum*, *G. raimondii*, and *G. arboreum*, we first collected the available genomic data for three cotton species from Cotton Functional Genomics Database (CottonFGD) [72]. Next, local databases were established for the nucleic acid and protein sequences of the predicted genes. The reported *A. thaliana*, *P. trichocarpa*, *O. sativa*, and *Z. mays* NHX protein sequences were downloaded from the published articles [46,73] (Table S5) and used as queries for identifying candidate sequences based on BlastP and tBlastN searches (default parameters) in the constructed local databases. The Pfam database was used to validate the candidate sequences and identify NHX genes in the cotton genomes [74].

### 4.2. Phylogenetic Relationship, Chromosomal Localization, Gene Structure, and Conserved Motif Analyses

The NHX protein sequences were aligned with the default multiple alignment parameters of Clustal W (http://www.clustal.org/clustal2/) [75]. The phylogenetic tree was constructed with the aligned sequences according to the neighbor-joining method of MEGA X (https://www.megasoftware.net/), with 1000 bootstrap replicates [76]. The chromosomal locations of *GhNHX* genes were obtained from the cotton genome database, and visualized by the TBtools program [77]. The Gene Structure Display Server (GSDS 2.0; http://gsds.cbi.pku.edu.cn/) was used to visualize the exon–intron structures of *GhNHX* genes based on genomic and coding sequences (Table S2) [78]. The conserved motifs in the GhNHX proteins (Table S2) were analyzed with the MEME software (version 5.1.1) (http://meme-suite.org/) [79].

### 4.3. Plant Materials and Treaments

Plump upland cotton TM-1 seeds were soaked in sterile water overnight in an incubator at 30 °C. The seeds with an exposed radicle tip were transferred to vermiculite, which was then covered with a plastic film to maintain humid conditions. The seeds were incubated at 23 °C and 60% humidity with a 16 h light/8 h dark cycle. Generally, the germinated seedlings were detected after 2–3 days, and the plastic film was removed. The seed coat was gently removed, and the cotyledon was flattened slightly. Then the seedlings were transferred to a hydroponic box and incubated for about 10 days (hoagland nutrient solution is used). In order to analyze the expression of *GhNHX* genes under salt stress, cotton seedlings that were strong and consistent in size were selected for treatment with 200 mM NaCl. Cotton seedlings treated with deionized water were used as mock. After 0, 1, 3, 6, and 12 h, the second true leaves were quickly frozen in liquid nitrogen and stored at −80 °C for total RNA extraction and reverse transcription.

### 4.4. RNA Isolation and Quantitative Real-Time PCR Analysis

Total RNA was isolated from each sample with the TRIzol reagent (TIANGEN, Beijing, China). The RNA concentration was determined with the NanoDrop 2000 nucleic acid analyzer (Thermo Fisher Scientific, USA), and the RNA quality was assessed by agarose gel electrophoresis. The first-strand cDNA was synthesized from 1 μg total RNA with the Transcriptor First-Strand cDNA Synthesis Kit (TaKaRa, Dalian, China). Regarding the qPCR, the *GhHIS3* gene was used as a reference control to normalize the cDNA amplification in each reaction. Moreover, the relative changes in the target gene expression levels were calculated with the 2^−ΔCt^ method [80]. The gene-specific primers used in this study are listed in Table S6. The cluster analysis, which was developed using the K-means method on the relative expression of *GhNHX* genes, was performed using the Genesis (version 1.8.1, Alexander Sturn and Rene Snajder, TU Graz) program.

### 4.5. Functional Analysis of GhNHXs Using Yeast Mutants

The NHX mutant yeast strain AXT3 (ena1−4Δ:HIS3, nha1Δ:LEU2, nhx1Δ:TRP1) derived from the wild-type yeast strain W303 is unable to transport Na^+^ and is therefore particularly sensitive to Na^+^. In contrast, yeast strain W303 (MAT: ura3-1, leu2-3, 112 his3-11, 15 trp1-1, ade2-1, can1-100) has a functional NHX protein that can transport Na^+^. The pYES2 vector, which is used for the galactose-induced expression of heterologous genes in yeast, contains an ampicillin-resistance gene and uracil (Ura) for selecting transformants. The *GhNHX1A* and *GhNHX4A* sequences were amplified (primer details are listed in Table S6) and then inserted into pYES2 to produce the pYES2-*GhNHX1A* and pYES2-*GhNHX4A* recombinant plasmids, respectively. Yeast strain AXT3 was separately transformed with pYES2, pYES2-*GhNHX1A,* and pYES2-*GhNHX4A*. Wild-type yeast strain W303 was used as the positive control. The W303, AXT3 transformed with pYES2-*GhNHX1A*, AXT3 transformed with pYES2-*GhNHX4A*, and AXT3 transformed with pYES2 were cultured in APG medium (10 mM arginine, 8 mM phosphoric acid, 2% glucose, 2 mM MgSO_4_, 1 mM KCl, 0.2 mM CaCl_2_, and trace minerals and vitamins). The effects of salt stress on yeast growth was assessed using APG medium (pH 6.5) with different concentrations of NaCl. Specifically, Ade, Ura, Trp, Leu, and His were added to APG medium, after which three salt stress treatment conditions were set: 0 (control), 30 mM NaCl, and 50 mM NaCl. When the precultures were supersaturated, they were diluted (20 times, 200 times, and 2000 times). An 8 µL aliquot of each diluted preculture was added to the prepared APG medium, which was then incubated at 30 °C for 3–5 days. Yeast growth was then analyzed and the colonies were photographed.

### 4.6. Transient Expression of Enhanced Green Fluorescent Protein (eGFP) Constructs in Tobacco Leaves

The *GhNHX4A* coding sequence was amplified from the *G. hirsutum* (cv TM-1) cDNA using Tks Gflex DNA Polymerase (Takara, Dalian, China). The *GhNHX4A* sequence (lacking a transcription termination codon) was then inserted into the *35S:eGFP* expression vector to generate the *35S:GhNHX4A-eGFP* recombinant plasmid for the subsequent expression of the eGFP-tagged fusion protein under the control of the CaMV 35S promoter. To ensure the expression construct was produced correctly, the recombinant plasmid was digested with specific restriction enzymes and sequenced. Details regarding the gene-specific primers are listed in Table S6.

Tobacco plump seeds were distributed evenly in the nutrient soil and incubated in an artificial climate room for about 1 week (20 °C and 60% humidity with a 16 h light/8 h dark cycle). From each bowl, a single healthy seedling was transferred to a new small bowl. Tobacco seedlings were grown until they produced approximately five leaves, after which they were used for the transient transformation experiment. First, 50 mL resistant Luria-Bertani culture was added to the culture comprising *A. tumefaciens* cells carrying the recombinant plasmid or the empty vector (control) in an ultra-clean workbench, and the culture was incubated at 28 °C with shaking (220 rpm) until the bacterial solution turned orange. The bacterial culture was centrifuged at 5000 rpm for 5 min. Resuspension solution (1 L resuspension requires 2.03 g of MgCl_2_, 2.135 g of MES monohydrate, and acetosyringone 0.03 g) was prepared, and the pelleted bacterial cells were resuspended for an OD_600_ value of 1.2. The resuspended bacterial solution was left standing at room temperature for 3 h in darkness. The third and fourth flat leaves from the top of tobacco plants were injected with the bacterial solution and marked. After a 24 to 72 h incubation in darkness, the leaf tissues near the injection site were cut and washed with deionized water. Then they were stained in the dark with 4 mM FM4-64 for 20–30 min and washed. Finally, they were analyzed with a laser scanning confocal microscope.

### 4.7. Virus-Induced Gene Silencing Analyses in Cotton

A *GhNHX4A* fragment was amplified and inserted into the VIGS vector (*TRV:00*) to produce the NHX-silencing construct. Details regarding the primers used are listed in Table S6. The *TRV:00* and *TRV:CLA* vectors were used as the negative and positive controls, respectively. *A. tumefaciens* GV3101 cells were separately transformed with the *TRV:GhNHX4A* recombinant plasmid as well as the control vectors. *TRV1* was mixed with *TRV:CLA*, *TRV:00,* and *TRV:GhNHX4A*, respectively. Then the cotyledon of 10-day-old cotton seedlings was infiltrated with a syringe. When the positive phenotype was observable, the roots, stems and leaves were collected to assess the effects of VIGS by qPCR analysis. The *TRV:00* and *TRV:GhNHX4A* plants were then used for treatment with 200 mM NaCl. And the other plants treated with deionized water were used as mock. After 10 days of the salt treatment, the *TRV:00* and *TRV:GhNHX4A* plants were photographed. And the roots, stems, and second true leaves of the *TRV:00* and *TRV:GhNHX4A* plants under the salt treat and mock were collected.

### 4.8. Determination of Intracellular K^+^ and Na^+^ Contents

The roots, stems, and second true leaves of the *TRV:00* and *TRV:GhNHX4A* plants were dried at 90 °C, and grounded to a powder. Next, a 0.05 g ground sample was dissolved in 5 mL concentrated HNO_3_ (i.e., nitrification). The solution was then diluted with deionized water 12 times and centrifuged. The supernatant was collected for an analysis of the ion content via atomic absorption spectroscopy.

## 5. Conclusions

In this study, 22 *G. hirsutum* NHX genes were identified and divided into two categories, with 18 and 4 in the vacuolar and endosomal groups, respectively. The gene structures and conserved motifs of the *G. hirsutum* NHX genes in the same phylogenetic cluster were relatively conserved during evolution. Additionally, the *G. hirsutum* NHX family expanded mainly via whole-genome duplication during the polyploidization event. Salt stress differentially up-regulated the expression of most of the *G. hirsutum* NHX genes. Interestingly, in the endosomal group, the expression of *GhNHX4A* was substantially up-regulated by salt stress. In addition, *GhNHX4A* can significantly restore the salt tolerance of yeast mutants. Moreover, the protein encoded by *GhNHX4A* was localized in the endosome, which was consistent with the results of phylogenetic analysis. Furthermore, the silencing of *GhNHX4A* considerably decreases the resistance of cotton to salt stress by increasing the accumulation of Na^+^ in stems and decreasing the accumulation of K^+^ in roots, implying that *GhNHX4A* may positively regulate the salt tolerance signaling pathway of cotton. The results of this study provide insights into the NHX functions in cotton, especially regarding the endosomal-type subfamily members. The data provided herein may be useful for future investigations of the molecular mechanism controlling cotton responses to salt stress. The genes characterized in this study may also be promising candidate genes for the breeding of new cotton varieties exhibiting increased salt tolerance.

## Supplementary Materials

The following are available online https://www.mdpi.com/1422-0067/21/20/7712/s1. Table S1. Details regarding *G. hirsutum* NHX genes. Table S2. Protein, coding and genomic sequences of *G. hirsutum* NHX genes. Table S3. Details regarding *G. raimondii* and *G. arboreum* NHX genes. Table S4. Protein sequences of G. *raimondii* and *G. arboreum* NHX genes. Table S5. Protein sequences of NHX genes from *A. thaliana, P. trichocarpa, O. sativa*, and *Z. mays*. Table S6. Primers used in this study. Figure S1. Functional complementation of *GhNHX1A* and *GhNHX4A* in yeast mutants. Figure S2. The expression of *GhNHX4D* in the *TRV:00* and *TRV:GhNHX4A* plants.

## Abbreviations

CPA1	cation/proton antiporter-1
NHX	Na+/H+ antiporter
qPCR	quantitative real-time PCR
VIGS	virus-induced gene silencing
