# Supplementary figures and images for: Genome-Wide Identification of the Gossypium hirsutum NHX Genes Reveals That the Endosomal-Type GhNHX4A Is Critical for the Salt Tolerance of Cotton
Source: Int J Mol Sci. 2020 Oct 18;21(20):7712. doi: 10.3390/ijms21207712 (PMC7589573; doi:10.3390/ijms21207712)

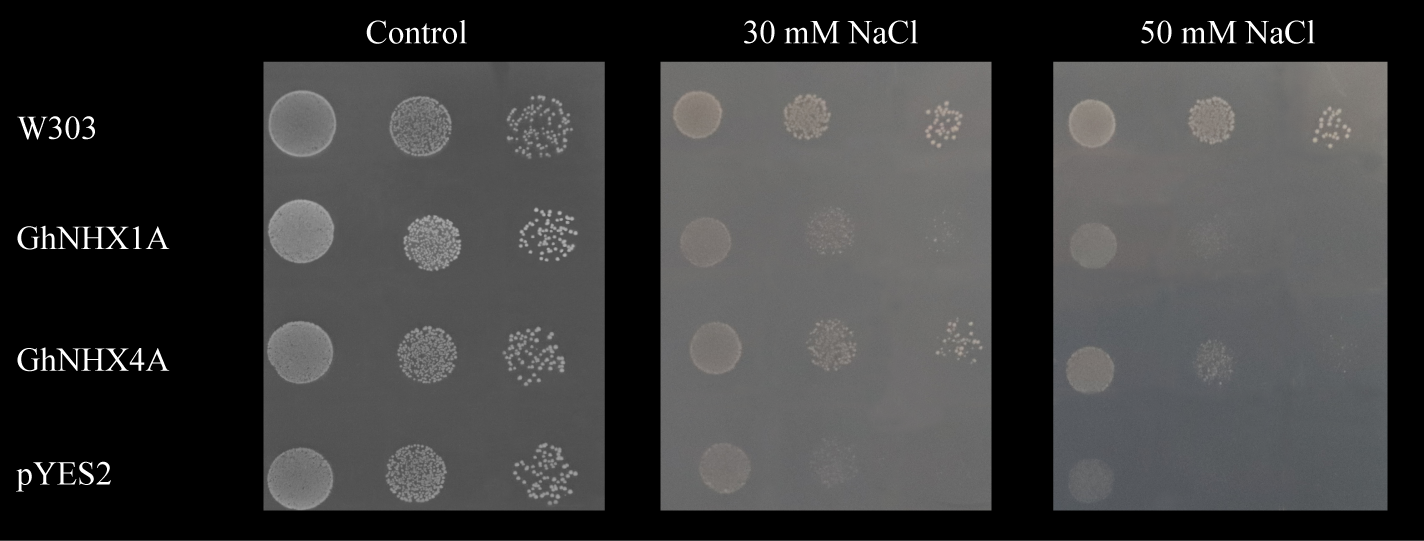

Supplement: Supplementary file 1 [file ijms-21-07712-s001.zip › Supplementary Materials/Figure S1.tif]

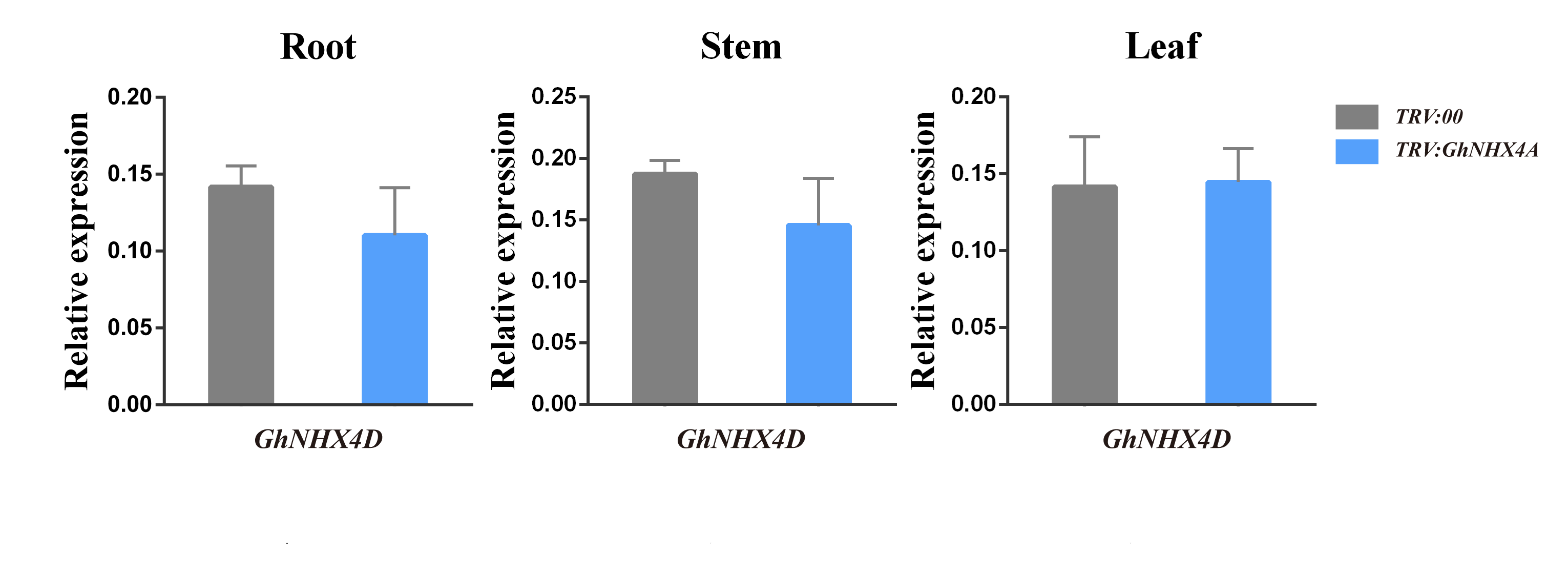

Supplement: Supplementary file 1 [file ijms-21-07712-s001.zip › Supplementary Materials/Figure S2.tif]
